# The BNT162b2 mRNA COVID-19 Vaccine Increases the Contractile Sensitivity to Histamine and Parasympathetic Activation in a Human Ex Vivo Model of Severe Eosinophilic Asthma

**DOI:** 10.3390/vaccines11020282

**Published:** 2023-01-28

**Authors:** Luigino Calzetta, Alfredo Chetta, Marina Aiello, Annalisa Frizzelli, Josuel Ora, Enrico Melis, Francesco Facciolo, Lorenzo Ippoliti, Andrea Magrini, Paola Rogliani

**Affiliations:** 1 Department of Medicine and Surgery, University of Parma, 43121 Parma, Italy; 2 Department of Experimental Medicine, University of Rome “Tor Vergata”, 00133 Rome, Italy; 3 Thoracic Surgery Unit, Regina Elena National Cancer Institute, 00144 Rome, Italy; 4 Department of Biomedicine and Prevention, University of Rome “Tor Vergata”, 00133 Rome, Italy

**Keywords:** airway hyperresponsiveness, asthma, BNT162b2, bronchospasm, comirnaty, COVID-19, exacerbation, eosinophils, isolated airways, polyethylene glycol, macrogol, safety, SARS-CoV-2, tozinameran, vaccine

## Abstract

The BNT162b2 COVID-19 vaccine is composed of lipid-nanoparticles (LNP) containing the mRNA that encodes for SARS-CoV-2 spike glycoprotein. Bronchospasm has been reported as an early reaction after COVID-19 mRNA vaccines in asthmatic patients. The aim of this study was to investigate the acute impact of BNT162b2 in a human ex vivo model of severe eosinophilic asthma. Passively sensitized human isolated bronchi were challenged with the platelet-activating factor to reproduce ex vivo the hyperresponsiveness of airways of patients suffering from severe eosinophilic asthma. BNT162b2 was tested on the contractile sensitivity to histamine and parasympathetic activation via electrical field stimulation (EFS); some experiments were performed after mRNA denaturation. BNT162b2 increased the resting tone (+11.82 ± 2.27%) and response to histamine in partially contracted tissue (+42.97 ± 9.64%) vs. the control (*p* < 0.001); it also shifted the concentration-response curve to histamine leftward (0.76 ± 0.09 logarithm) and enhanced the response to EFS (+28.46 ± 4.40%) vs. the control. Denaturation did not significantly modify (*p* > 0.05) the effect of BNT162b2. BNT162b2 increases the contractile sensitivity to histamine and parasympathetic activation in hyperresponsive airways, a detrimental effect not related to the active component but to some excipient. A possible candidate for the bronchospasm elicited by BNT162b2 could be the polyethylene glycol/macrogol used to produce LNP.

## 1. Introduction

Asthma is a heterogeneous disease characterized by chronic airway inflammation associated with respiratory symptoms and variable expiratory airflow limitation [1]. Severe asthmatic patients are at high risk of airway hyperresponsiveness (AHR) in response to several stimuli, mainly due to the activation of resident eosinophils in the bronchial tissue leading to bronchospasm [2,3,4]. Asthmatic patients experiencing ≥3 exacerbations per year may have a distinct frequent asthma exacerbation phenotype. Such a condition is called exacerbation-prone asthma (EPA) with specific implications for the targeting of exacerbation prevention strategies [2].

BNT162b2, the most widely administered COVID-19 vaccine in Europe, is composed of lipid-nanoparticles (LNP) containing the mRNA that encodes for the SARS-CoV-2 spike glycoprotein [5,6]. The rate of allergic reactions to COVID-19 mRNA vaccines is greater among patients suffering from a history of high-risk allergies. Generally, COVID-19 vaccination has been recommended for severe asthmatics, as the reported adverse effects consisting of acute bronchospasm are rare, but possible [7,8,9]. In severe asthmatic patients treated with biologic therapies such as benralizumab, mepolizumab, and dupilumab, conflicting findings resulted from real-world settings concerning the response to the COVID-19 vaccine: some studies reported significantly lower antibody levels compared with healthy subjects after vaccination, whereas other studies reported no significant difference in antibody response to the vaccine compared to other patient groups after either two or three vaccine doses [10,11]. In any case, it seems that the type of biological treatment does not affect vaccine-elicited humoral and cellular immunity in severe asthmatic patients [12].

Although it has been suggested that most patients with a history of allergic diseases can be safely immunized with COVID-19 mRNA vaccines, significant bronchospasm has been reported as an early reaction within 10 to 20 min after the first dose of the vaccine in asthmatic patients [5,13]. As a matter of fact, in these subjects, a 30-min observation period is suggested after receiving COVID-19 mRNA vaccines to check for potential allergic reactions [14].

Certainly, it is striking that more than 80% of the reported anaphylaxis cases due to mRNA vaccines occurred primarily after the first dose administration [15]. Indeed, these epidemiological data support a possible link between COVID-19 mRNA vaccination and asthma exacerbation, especially in severe patients suffering from EPA [16,17]. However, no data are currently available regarding a potential effect of BNT162b2 on human airway smooth muscle (ASM). Despite the claimed favorable safety profile of BNT162b2, adverse events were reported only generically in the primary publication with no specific frequencies [18]. Unexpectedly, after more than two years from the publication of data [18], detailed results regarding the real safety profile of BNT162b2 are not available on the ClinicalTrials.gov database (NCT04368728). In addition, real-world safety data are scarce [19].

In the light of this evidence, we have hypothesized that BNT162b2 may have a detrimental effect on the airways of patients suffering from severe asthma, who are characterized by baseline AHR and activated eosinophils infiltrating the bronchial tissue [20]. Therefore, the aim of this study was to investigate the acute impact of BNT162b2 on AHR in a human ex vivo model of severe eosinophilic asthma.

## 2. Materials and Methods

### 2.1. Tissue Collection and Preparation

Regions of lungs were taken from uninvolved areas of neoplastic lesions and resected from 10 patients undergoing lobectomy surgery for lung cancer. Tissue was placed in Krebs–Henseleit (KH) buffer solution (NaCl, 119.0 mmol; KCl, 5.4 mmol; CaCl_2_, 2.5 mmol; KH2PO4 mmol, 1.2 mmol; MgSO_4_, 1.2 mmol; NaHCO_3_, 25.0 mmol; glucose, 11.7 mmol; pH 7.4) containing indomethacin (5 μM) and transported to the Laboratory of Respiratory Diseases at the University of Rome “Tor Vergata” (Italy) from a nearby hospital.

All the donors did not suffer from chronic obstructive respiratory disorders, including asthma and chronic obstructive pulmonary disease. None of the patients had been chronically treated with corticosteroids and/or bronchodilators, and serum immunoglobulin E (IgE) levels were in the normal range (<100 IU/mL). Additionally, preoperative lung function parameters were in the normal range. Detailed demographic and metric characteristics of donors are reported in Table 1. In the laboratory, the airways were cut into rings (sub-segmental bronchi: thickness 1–2 mm, diameter 4–5 mm) [21].

### 2.2. Passive Sensitization and Eosinophils Activation

The passive sensitization is a model that closely mimics important functional characteristics of non-specific AHR in asthmatic patients, as previously reported [23,24]. Isolated airways were rotated overnight at room temperature in tubes containing KH buffer solution in the presence of 10% vol^−1^ sensitizing serum or 10% vol^−1^ non-sensitizing serum. Sensitizing and non-sensitizing sera were prepared by centrifugation from the whole blood. Sensitizing serum was obtained from a patient suffering from atopic asthma (total IgE > 1000 U mL^−1^ specific against common aeroallergens) during an exacerbation, whereas non-sensitizing serum was obtained from a non-atopic donor (total IgE 48 U mL^−1^). The subjects provided signed consent for serum donation. Sera were frozen at −80 °C in 250 µL aliquots until required. The day after, before beginning the experiments, passively sensitized airways were challenged for 45 min with platelet-activating factor (PAF, 100 nM), a procedure that activates resident eosinophils normally present in the bronchial tissue [25,26,27]. Overall, challenging passively sensitized human isolated airways with PAF is a validated method that reproduces ex vivo important characteristics of AHR in severe eosinophilic asthma [24,25,28,29,30,31].

### 2.3. Transmural Stimulation

Transmural stimulation, also called electrical field stimulation (EFS), was performed by placing tissues between two wire platinum electrodes (20 mm apart, Panlab Harvard Apparatus, Barcelona, Spain) connected to a 3165 multiplexing pulse booster stimulator (Ugo Basile, Gemonio, Italy). Bronchial rings were contracted by EFS at increasing frequencies (EFS_3–25Hz_, 10 V, 10 s, 0.5 ms, biphasic pulse) in order to stimulate the resident parasympathetic ganglia to activate the vagus nerve firing (parasympathetic pathway) observed in human in vivo and, thus, eliciting a contractile response via the release of endogenous acetylcholine [32].

### 2.4. Preparation of Drugs

The BNT162b2 vaccine (Pfizer-BioNTech, New York, NY, USA) includes the active component tozinameran, a highly purified single-stranded 5′-mRNA encoding the viral spike protein of SARS-CoV-2, and several excipients, such as polyethylene glycol/macrogol (PEG) as part of ALC-0159 (https://www.gov.uk/government/publications/regulatory-approval-of-pfizer-biontech-vaccine-for-covid-19/information-for-healthcare-professionals-on-pfizerbiontech-covid-19-vaccine, accessed on 21 December 2022). BNT162b2 was stored according to the fact sheet (https://www.pfizer.com/products/product-detail/pfizer-biontech-covid-19-vaccine, accessed on 21 December 2022) and used immediately after being diluted in its specific diluent (sodium chloride 0.9% solution for injection, vehicle). Residual volumes of diluted vaccine collected from those vials from which it was not possible to extract the extra 6th dose were used in this study (https://www.ema.europa.eu/en/news/extra-dose-vials-comirnaty-covid-19-vaccine, accessed on 21 December 2022).

Histamine (Sigma-Aldrich, Milan, Italy) was diluted in distilled water and indomethacin (Sigma-Aldrich, Milan, Italy) in dimethyl sulfoxide (DMSO). The maximum amount of ethanol and DMSO did not influence the isolated tissue response [33]. Histamine and indomethacin were stored in small aliquots at −80 °C until their use.

### 2.5. Contraction Measurement

Bronchial rings were mounted into a 10-mL High Tech 8 Channels Manual Compact Organ Bath system (Panlab Harvard Apparatus, Barcelona, Spain) containing KH buffer solution (37 °C) aerated with O_2_/CO_2_ (95:5%) and attached to isometric force transducers Fort25 (WPI, Middlewich, UK). Isolated airways were allowed to equilibrate by flushing with fresh KH buffer solution. Passive tension was determined by the gentle stretching of tissue (0.5–1.0 g) during equilibration. When the passive contractile tone reached the plateau, bronchial rings were washed three times with KH buffer solution and allowed to further equilibrate for 45 min.

### 2.6. Study Characteristics

This study was ex vivo, prospective, randomized, controlled, and blinded, with parallel groups.

### 2.7. Endpoint

The endpoint of this study was to assess the impact of BNT162b2 on the contractile sensitivity to histamine and parasympathetic activation in a human ex vivo model of severe eosinophilic asthma.

### 2.8. Study Design

Concentration-response curves (CRCs) to BNT162b2 were constructed on the resting tone of isolated airways and on bronchial tissue partially pre-contracted by histamine administered at concentrations eliciting 20% the maximal effect (EC_20_); each next drug administration was performed when the previous administered concentration elicited a contractile plateau, generally 5–15 min. The effect of pre-treating 45 min isolated airways with BNT162b2 was also tested on the CRC to His. Further bronchial tissue was stimulated by EFS_3–25Hz_, with each EFS delivered after that the contractile response induced by the previous EFS was terminated, usually after 3–5 min. Experiments were performed in both non-sensitized (NS) bronchial tissue and in passively sensitized plus PAF challenged (sens+PAF) isolated airways; the vehicle was used as a control. In order to assess the component of the vaccine (active component vs. excipients) which is potentially responsible for AHR, some experiments were performed by using BNT162b2 after mRNA denaturation (heated 5 min at 70 °C), a condition that does not alter the PEG structure [34,35].

### 2.9. Pharmacological and Statistical Analysis

In experiments performed on resting and histaminergic tone, results were reported as the percentage of the maximal effect (E_max_) induced by histamine [36]; in experiments performed on EFS_3–25Hz_, results were reported as the percentage of E_max_ elicited by 25 Hz in control bronchi [37]. Appropriate curve-fittings to sigmoidal models were used to calculate the half maximal effective concentration (EC_50_). The equations used to describe the models were Y = Bottom + (Top − Bottom)/(1 + 10^((LogEC_50_ − X) × HillSlope)) and Y = Bottom + (Top − Bottom)/(1 + 10^((LogEC_50_ − X))), according to the goodness of fit. EC_50_ was transformed into pEC_50_ (−LogEC_50_) to perform the statistical analysis of the potency [38].

Results were reported as mean ± standard error (SE), with two-way analysis of variance (ANOVA) with multiple comparisons used to assess the level of statistical significance for *p* < 0.05.

### 2.10. Sample Size

No published data are currently available concerning the impact of BNT162b2 on AHR in human airways; therefore the sample size for this study cannot be calculated. However, n ≥ 5 has been used according to the statistical reporting guidelines for pharmacological studies [39].

## 3. Results

### 3.1. Baseline Characteristics

No significant difference resulted for the stability of resting tone between NS and sens+PAF (*p* > 0.05). Sens+PAF isolated airways were significantly (*p* < 0.01) hyperresponsive to histamine compared to NS isolated airways (delta pEC_50_: 0.34 ± 0.08). The baseline contractile response to histamine is reported in Table 2. Sens+PAF bronchi where also significantly (*p* < 0.05) hyperresponsive to EFS compared to NS bronchi (720 ± 140 mg vs. 410 ± 90 mg, respectively).

### 3.2. Effect of BNT162b2 on Isolated Airways Resting Tone

**Figure 1:**
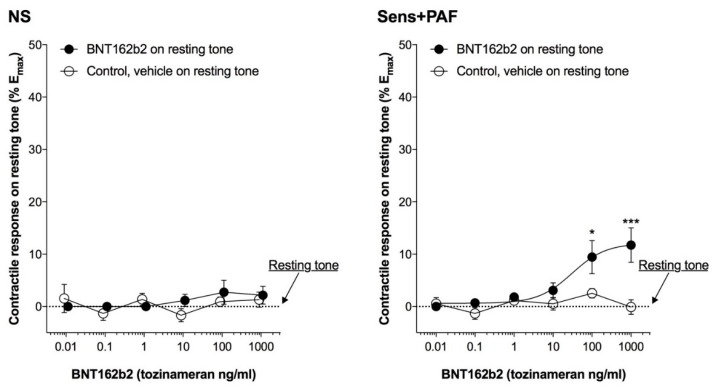
Effect of BNT162b2 on isolated airways resting tone in NS and sens+PAF isolated airways. * *p* < 0.05 and *** *p* < 0.001 vs. control; results reported as mean ± SE of n = 5 human isolated sub-segmental bronchi. NS: non-sensitized; PAF: platelet-activating factor; SE: standard error; sens+PAF: passively sensitized plus PAF challenge.

BNT162b2 100–1000 ng/mL slightly but significantly increased the resting tone in sens+PAF isolated airways (E_max_ +11.82 ± 2.27%, *p* < 0.001 vs. control) but not in NS isolated airways (*p* > 0.05 vs. control) (Figure 1).

### 3.3. Effect of BNT162b2 on Isolated Airways Partially Pre-Contracted by Histamine

**Figure 2:**
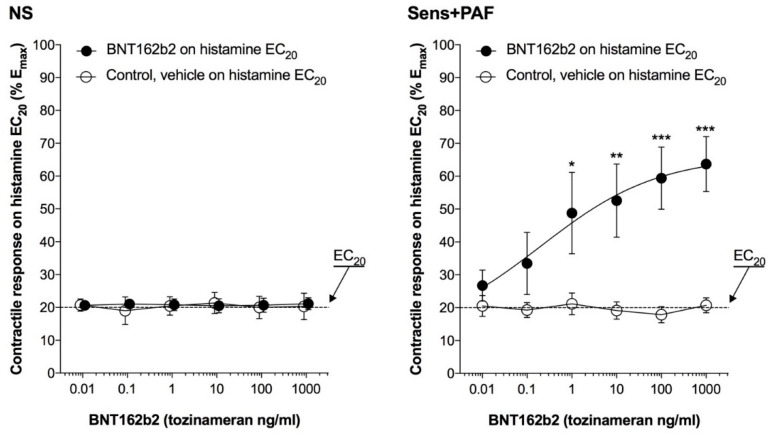
Effect of BNT162b2 in NS and sens+PAF isolated airways partially pre-contracted by histamine. * *p* < 0.05, ** *p* < 0.01, and *** *p* < 0.001 vs. control; results reported as mean ± SE of n = 5 human isolated sub-segmental bronchi. EC_20_: concentrations eliciting 20% the maximal effect; NS: non-sensitized; PAF: platelet-activating factor; SE: standard error; sens+PAF: passively sensitized plus PAF challenge.

BNT162b2 1–1000 ng/mL significantly increased the contractile response to histamine administered at EC_20_ in sens+PAF isolated airways (E_max_ +42.97 ± 9.64%, *p* < 0.001 vs. control) but not in NS isolated airways (*p* > 0.05 vs. control) (Figure 2). The pEC_50_ of BNT162b2 on sens+PAF isolated airways partially pre-contracted by histamine was 0.52 ± 0.75.

### 3.4. Effect of BNT162b2 on the CRC to Histamine

**Figure 3:**
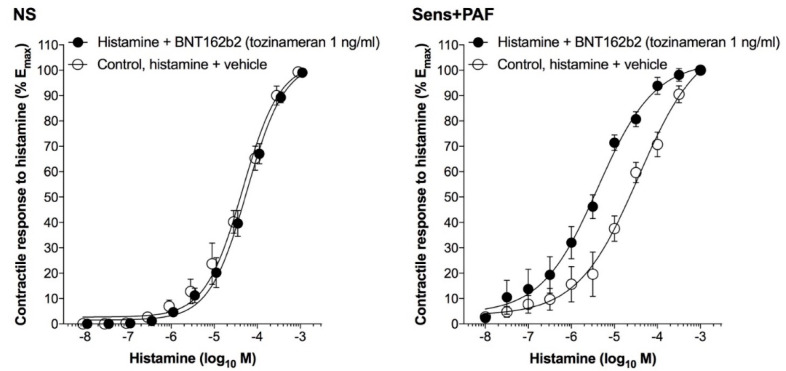
Effect of BNT162b2 on the CRCs to histamine in NS and sens+PAF isolated airways. Results reported as mean ± SE of n = 5 human isolated sub-segmental bronchi. CRC: concentration-response curve; NS: non-sensitized; PAF: platelet-activating factor; sens+PAF: passively sensitized plus PAF challenge.

In sens+PAF airways, BNT162b2 1 ng/mL significantly shifted leftward the CRC to histamine by 0.76 ± 0.09 logarithm (*p* < 0.001 vs. control); BNT162b2 1 ng/mL did not significantly modify the CRC to histamine in NS isolated airways (*p* > 0.05 vs. control) (Figure 3 and Table 2).

### 3.5. Effect of BNT162b2 on EFS_3–25Hz_

**Figure 4:**
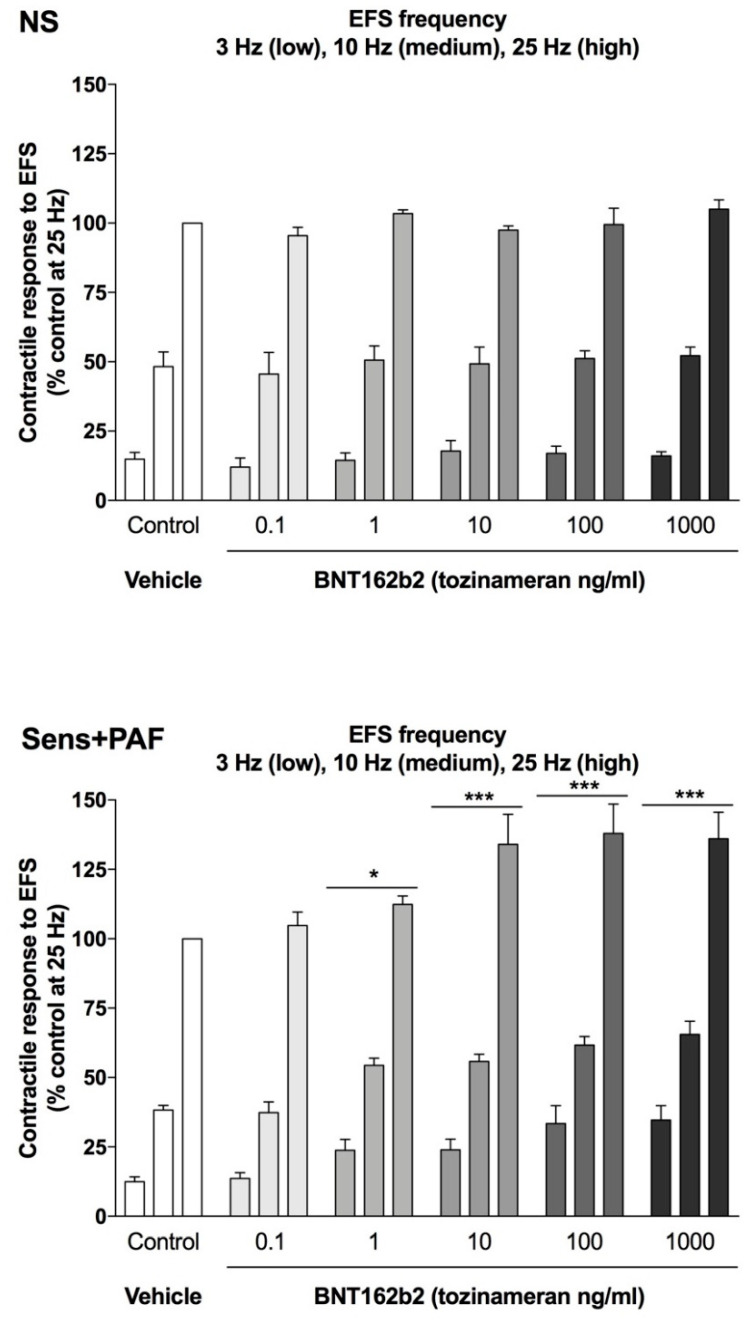
Effect of BNT162b2 on the parasympathetic contractile response induced by EFS_3–25Hz_ in NS and sens+PAF isolated airways. * *p* < 0.05 *** *p* < 0.001 vs. control; results reported as mean ± SE of n = 5 human isolated sub-segmental bronchi. EFS: electrical field stimulation; NS: non-sensitized; PAF: platelet-activating factor; SE: standard error; sens+PAF: passively sensitized plus PAF challenge.

**Figure 5:**
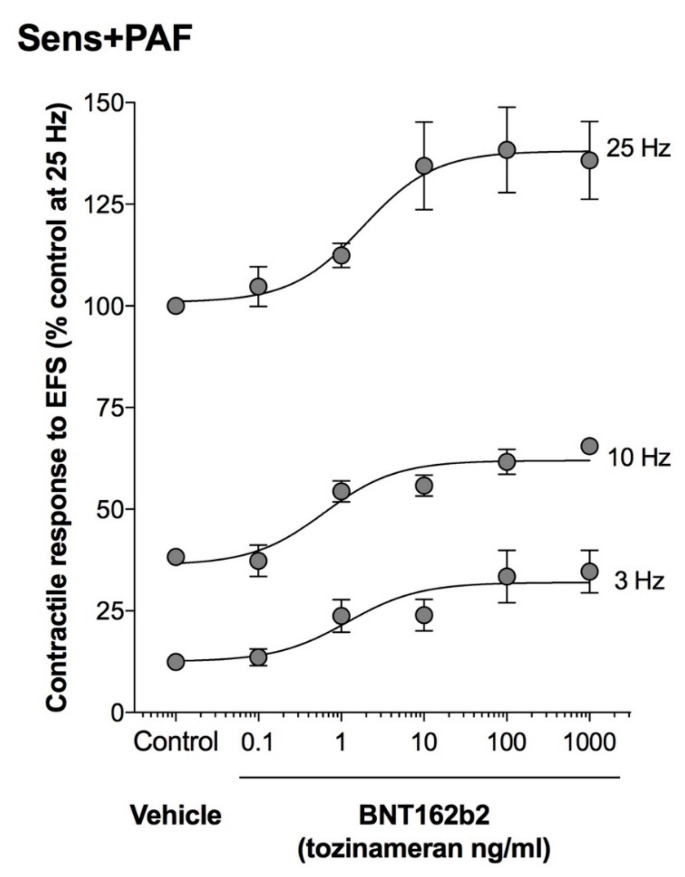
CRCs to BNT162b2 on the parasympathetic contractile response induced by EFS_3–25Hz_ in sens+PAF isolated airways. Results reported as mean ± SE of n = 5 human isolated sub-segmental bronchi. EFS: electrical field stimulation; PAF: platelet-activating factor; SE: standard error; sens+PAF: passively sensitized plus PAF challenge.

BNT162b2 1–1000 ng/mL significantly enhanced the contractile response to EFS_3–25Hz_ in sens+PAF isolated airways (overall E_max_ +28.46 ± 4.40%, *p* < 0.001 vs. control) but not in NS isolated airways (*p* > 0.05 vs. control) (Figure 4). The pEC_50_ of BNT162b2 on EFS_3–25Hz_ was not significantly (*p* > 0.05) related to the frequency and resulted to be 1.19 ± 1.24 at 3 Hz, 0.62 ± 0.34 at 10 Hz, and 1.83 ± 1.78 at 25 Hz (Figure 5).

### 3.6. Effect of Denatured BNT162b2 on the CRC to Histamine

The mRNA denaturation did not significantly modify (*p* > 0.05) the effect of BNT162b2 1 ng/mL on the contractile response to histamine (Table 2) and EFS_3–25Hz_ (overall delta effect: −4.60 ± 4.45% vs. not denatured, data not shown).

## 4. Discussion

The evidence provided by this study indicates that the mRNA COVID-19 vaccine BNT162b2 increases the contractile sensitivity to histamine and parasympathetic activation in passively sensitized human isolated airways challenged with PAF, a condition reproducing the AHR typical of severe eosinophilic asthma ex vivo. Conversely, BNT162b2 did not alter the contractile response to histamine and the parasympathetic activation in the control bronchial tissue. Specifically, in airways undergoing severe eosinophilic asthma model, BNT162b2 increased the potency of histamine by almost one logarithm and enhanced the contractile response to EFS by around 30%, regardless of the frequency delivered. Interestingly, these effects were unrelated to the denaturation of vaccine mRNA, a procedure that does not alter the structure of the LNP in which the mRNA is encapsulated [34,35,40]. Therefore, the increased contractility to BNT162b2 in hyperresponsive airways was not correlated to the active component tozinameran but to excipients. Although identifying the component within the vaccine excipients leading to bronchospasm is out of the scope of this study and needs yet to be determined, it seems that the PEG used to produce the LNP could be a possible candidate [40,41].

BNT162b2 includes the following lipid components: ALC-0315, 1,2-distearoyl-sn-glycero-3-phosphocholine, cholesterol, and ALC-0159 at a 46.3:9.4:42.7:1.6 molar ratio (%). Additionally, other excipients are included, such as potassium, chloride, potassium dihydrogen phosphate, sodium chloride, disodium hydrogen phosphate dihydrate, sucrose, and water, for injections [40,42]. Among the reported excipients, the one with the ability to cause bronchospasm is ALC-0159 since it contains PEG, a component related to an increasing number of allergic reactions in the last years [42,43,44,45]. Since PEG is a high-risk hidden allergen in drugs inducing allergic reactions that are difficult to detect, it might be underdiagnosed [46]. Certainly, our findings support the increasing body of evidence that BNT162b2 may induce allergic reactions and bronchospasm after the first dose administration, with ALC-0159 being the most probable cause [5,15,42].

The consistent effect of BNT162b2 on both histaminergic and parasympathetic pathways suggests that the vaccine may both directly and indirectly increase AHR by acting on ASM, vagal fibers, and intramural ganglia [47,48]. Considering the effect on all the EFS frequencies investigated in this study, evidently BNT162b2 may have a facilitator activity on the release of contractile neurotrasmitters from all the parasympathetic post-ganglionic neurons, including the large and small myelinated A- and B-fibers, as well as the nonmyelinated C-fibers, the latter of which are involved in the acute neurogenic inflammation that is a detrimental condition in severe asthma [49,50]. Of note, although moderately and at higher concentrations, BNT162b2 increased the resting tone of isolated airways; moreover, it significantly shifted the contractile response upward in partially pre-contracted airways.

Indeed, these findings may have some relevant clinical implications that can be easily interpreted. In fact, the evidence that BNT162b2 does not impair ASM contractility in control isolated airways supports the favorable respiratory safety profile of this vaccine when administered to the general population, as previously reported from randomized controlled trials (RCT) [18,51]. On the other hand, the detrimental influence of BNT162b2 on airway contractility in the model of severe eosinophilic asthma should be carefully assessed in asthmatic populations by considering the levels of concentrations and the biodistribution of the vaccine after administration.

According to the BNT162b2 Assessment Report provided by the European Medicine Agency [52], after administration LNP can be detected in most tissues, including airways, with the injections site and the liver as the sites of major biodistribution. Considering that the amount of ALC-0159 excipient in the finished product is 50 μg/dose and that this LNP has a low affinity for tissue [52], it should be assessed whether very low concentrations of the vaccine might be able to elicit bronchospasm in sensitive populations, such as asthmatic patients after first administration. Unfortunately, currently data on the biodistribution at the level of the respiratory system are not officially available; moreover, serious concerns have been raised whether adequate safety testing for BNT162b2 were performed in preclinical trials, including pharmacokinetic (PK) studies [52,53,54]. However, an independent PK study carried out in mice [55] showed that PEG-nanoparticles can be significantly detected in the lungs, reaching up to 4% of the injected dose. Therefore, considering that the average weight of both lungs including airways is around 840 g in men [56], it can be estimated that concentrations >2.38 ng/mL of ALC-0159 can be detected in the bronchial tissue after administration, according to the following formula: [(ALC-0159 ng/dose: 50,000) × (lung biodistribution: 4%)/(lung weight: 840 g)]. Since in women the average weight of both lungs including airways is around 640 g, the concentrations of ALC-0159 can be estimated to be >3.12 ng/mL [57]. Indeed, in children and adolescents receiving BNT162b2, the estimated concentrations of ALC-0159 at the level of the respiratory system would be linearly greater.

The results of this study have been reported by anchoring the dilution of BNT162b2 with the levels of concentrations of tozinameran. Since the ratio between ALC-0159 and tozinameran is 5:3 (ALC-0159 50 μg/dose vs. tozinameran 30 μg/dose) [40,52], each ng/mL of tozinameran corresponds to 1.66 ng/mL of ALC-0159. Thus, according to the results of this investigation and the estimated concentrations of ALC-0159 in the respiratory system, it is possible that even the very low concentrations of ALC-0159 administered through a single BNT162b2 injection may elicit bronchospasm in subjects at risk, such as asthmatic patients. Indeed, this translational evidence should be taken into account in the daily clinical practice of COVID-19 vaccination.

The main limitation of this research is intrinsic to the ex vivo experimental approach and, thus, results originating from the model of severe eosinophilic asthma need to be translated into real-life. Additionally, the use of tissue from donors undergoing lobectomy surgery for cancer may represent a potential bias compared to performing experiments using tissue from individuals that had no lung cancer. However, data obtained from ex vivo pharmacological studies have usually been confirmed in clinical settings [58,59,60,61,62]. From a clinical point of view, it is not possible to check if BNT162b2 may really elicit a greater risk of bronchospasm compared to a placebo because, unexpectedly, the key sponsored phase 3 RCT on BNT162b2 (NCT04368728, NCT04713553, NCT04816669), although already completed and/or published in MEDLINE, reported no results posted in the ClinicalTrial.gov database [63,64]. This is a missing opportunity to assess the real safety profile concerning serious adverse events of BNT162b2 via pooled analysis, as already demonstrated in other scientific contexts [65,66]. Therefore, unbiased and well performed observational studies may help to solve this matter and confirm the findings of our research. Another limitation of our study is that each specific lipid excipient (i.e., ALC-0315, 1,2-distearoyl-sn-glycero-3-phosphocholine, cholesterol, and ALC-0159) was now investigated on ASM contractility. Finally, but not less important, we cannot exclude that the increased AHR elicited by BNT162b2 could also be related to the nanostructure of LNP, as previously demonstrated for carbon nanotubes [37].

## 5. Conclusions

As predicted by the hypothesis of this study, here we have demonstrated that BNT162b2 may elicit bronchospasm in hyperreactive airways, and that this detrimental effect results after a single administration, thus with no involvement of allergic sensitization mechanisms due to repeated exposures [67]. Certainly, we have ruled out that the increased AHR could be related to the active component tozinameran; conversely, we have proven that it is associated with some excipient, perhaps ALC-0159. Since asthmatic patients, especially children and adolescents, might be at higher risk for severe bronchospasm after BNT162b2 injection, a pre-treatment with an inhaled corticosteroid/formoterol combination before the administration of any vaccine containing LNP may help to protect against such a serious and potentially life-threating respiratory adverse event [68]. However, future research is needed to confirm this latter possibility.

## Figures and Tables

**Table 1:** Demographic characteristics of human subjects and normal ranges, according to GOLD and GINA recommendations [1,22].

Characteristics	Tissue Used as Ex Vivo Model of Severe Eosinophilic Asthma	Normal Range
Gender (male/female)	5/5	/
Age (years)	53.5 ± 3.1	/
Height (cm)	168.2 ± 2.3	/
Weight (Kg)	67.4 ± 2.4	/
BMI	23.8 ± 0.8	18.5–24.9
Smoking status		
current	0	/
former	10	/
never	0	/
IgE	45.3 ± 6.4	<100
Pack years	16.5 ± 2.1	/
FEV_1_ (L)	2.75 ± 0.12	/
FEV_1_ (% predicted)	88.0 ± 3.8	>80
FEV_1_ reversibility (%)	4.8 ± 1.1	<12%
FVC (L)	3.58 ± 0.15	/
FEV_1_/FVC	0.77 ± 0.01	>0.7

Results reported as mean ± SE of n = 10 subjects. BMI: body mass index; FEV_1_: forced expiratory volume in 1 s; FVC: forced vital capacity; GINA: Global Initiative for Asthma; GOLD: Global Initiative for Chronic Obstructive Lung Disease; IgE: immunoglobulin E; IU: international units; SE: standard error.

**Table 2:** Effect of BNT162b2 and mRNA denaturation on the pharmacological characteristics of CRC to histamine in NS and sens+PAF isolated airways.

	NS		Sens+PAF	
	E_max_	pEC_50_	E_max_	pEC_50_
Control, histamine + vehicle	103.10 ± 3.56	4.29±0.07	96.57 ± 3.63	4.63 ± 0.09 **
Histamine + BNT162b2 1 ng/ml	101.30 ± 2.71	4.27±0.05	97.20 ± 2.73	5.39 ± 0.09 *** ^§^
Histamine + denatured BNT162b2 1 ng/ml	/	/	101.0 ± 6.10	5.25 ± 0.14 ^§^

**: *p* < 0.01 and ***: *p* < 0.001 vs. NS; ^§^: *p* < 0.001 vs. control; results reported as mean ± SE of n = 5 human isolated sub-segmental bronchi. /: experiments not performed; CRC: concentration-response curve; EC_50_: half maximal effective concentration; E_max_: maximal effect; pEC_50_: −LogEC_50_: NS: non-sensitized; PAF: platelet-activating factor; SE: standard error; sens+PAF: passively sensitized plus PAF challenge.
